# 

**Published:** 1908-08-15

**Authors:** 


CONTENTS

Event and Comment - - - - - - - - 379

A Plea for Keener Observation, More Conservatism and a Better Understanding of the Question, "Which is the Best Alloy?" *By Marcus L. Ward, D.D.Sc.* 381

President's Address, - - - - *By Dr. Oel E. Lanphear* 406

Management of Putrescent Pulp and a Permanent Root Filling, *By Dr. T. C. Reid* 414

Value of Diagnosis in Filling Teeth, - *By J. V. Couzett, D.D.S.* 416

Indents - - - - - - - - - - - - - 422

Announcements - - - - - - - - - 428

